# Oncogenic mutations produce similar phenotypes in *Drosophila* tissues of diverse origins

**DOI:** 10.1242/bio.20147161

**Published:** 2014-02-25

**Authors:** Stefanie Stickel, Tin Tin Su

**Affiliations:** 1 Department of Molecular, Cellular and Developmental Biology, University of Colorado, Boulder, CO 80309-0347, USA; 2 University of Colorado Cancer Center, Aurora, CO 80045, USA

**Keywords:** *Drosophila*, Ras, Tumor, Cell cycle, Checkpoints, Aneuploidy

## Abstract

An emerging interest in oncology is to tailor treatment to particular cancer genotypes, i.e. oncogenic mutations present in the tumor, and not the tissue of cancer incidence. Integral to such a practice is the idea that the same oncogenic mutation(s) produces similar outcomes in different tissues. To test this idea experimentally, we studied tumors driven by a combination of *Ras^V12^* and *scrib^1^* mutations in *Drosophila* larvae. We found that tumors induced in tissues of neural ectodermal and mesodermal origins behaved similarly in every manner examined: cell cycle checkpoints, apoptosis, cellular morphology, increased aneuploidy and response to Taxol. We conclude that oncogenic effects override tissue-specific differences, at least for the mutations, tissues, and phenotypes studied herein.

## INTRODUCTION

Traditional cancer therapy is tailored to the site of disease (ovary, lung, brain, etc.). For example, a typical chemotherapy regime for ovarian cancer is cis/carboplatin plus a taxane, whereas a typical chemotherapy regime for colon cancer is oxaliplatin plus 5-FU (http://www.cancer.org). Development of therapies based on the disease site is also seen for targeted agents. For example, the EGFR inhibitor Erbitux is FDA-approved for colorectal and head and neck cancers, despite the fact that EGFR hyper-activation occurs in many cancer types. Recent advances in genome sequencing reveal common mutations in tumors from distinct tissue types. Consequently, an emerging interest in oncology is to tailor the treatment not to tissue origin but to oncogenic mutations present in the tumor. B-RAF inhibitors, for example, may be used on tumors with the cognate B-RAF mutation regardless of whether they are melanoma or colorectal cancer. Integral to such a practice is the idea that the same oncogenic mutation(s) produces similar outcomes in different organs. This hypothesis has never been tested experimentally. Indeed, one could envision two extreme possibilities. Oncogenic mutations may exert their effect regardless of cell type, producing similar outcomes. Alternatively, underlying differences amongst tissues (e.g. epigenetic status, transcription program or signaling pathways) could interact with the effects of oncogenic mutations in such a way to yield varying outcomes.

To compare the consequences of oncogenic mutations in different tissues, we utilized a well-characterized system to induce tissue-specific tumors in *Drosophila melanogaster* using oncogenic Ras (Brumby and Richardson, 2003; Dow et al., 2008; Humbert et al., 2008; Pagliarini et al., 2003). Activating mutations in the small GTPase Ras are found in 20–30% of human cancers, with *Ras^V12^* being the most frequent allele. Oncogenic mutants of Ras have been expressed in mice before (reviewed by Kamata and Pritchard, 2011), but these experiments used different alleles and different isoforms (KRas, NRas or HRas), precluding a direct comparison. In *Drosophila* larvae, ectopic expression of *Ras^V12^* induces overgrowth (Brumby and Richardson, 2003; Pagliarini et al., 2003). A screen for modifiers identified genes that normally control cellular apical–basal polarity including *scribble*. *scrib* encodes a homolog of the human tumor suppressor hScrib (Dow et al., 2003; Humbert et al., 2008). *scrib* homozygous mutant clones in larval imaginal discs are normally eliminated (Brumby and Richardson, 2003). However, when *scrib* mutations are combined with *Ras^V12^*, the resulting cells not only overgrew but also became invasive. In these studies, *eyeless* promoter-driven FLP recombinase (*eyFLP*) was used to target *Ras^V12^*, a GFP marker, and loss of heterozygosity (LOH) in *scrib* by mitotic recombination to the developing eye-antennae discs and the optic lobes. The resulting overgrowth invades the adjacent ventral nerve cord. Due to their accelerated growth rate that is no longer coordinated with the developmental program, these tumors have been called ‘neoplastic’ (Brumby and Richardson, 2003). Neoplastic tumors prolong the larval state for up to 13 days after egg deposition (AED), instead of the normal 5 days. Tumor-bearing larvae die without forming pupae. When transplanted into the abdomen of wild-type adult females hosts, neoplasms remained not only proliferative but also invasive, spreading into the intestines and the ovaries (Pagliarini and Xu, 2003).

To study tumors induced by the *Ras^V12^/scrib^1^* combination in different tissues, we took advantage of observations made in earlier studies, that in addition to tumors in the eye-antennae disc and the optic lobe of the brain (collectively referred to as cephalic tumors), some *eyFLP* drivers produced additional GFP-positive growths (Pagliarini and Xu, 2003). Here, we identified a mesoderm-derived cell population in the gonad as progenitors of secondary tumors, and compared the characteristics of cephalic and gonadal tumors. Our results support the hypothesis that oncogenic mutations can exert similar effects in diverse organs.

## RESULTS AND DISCUSSION

### Over-growth in the gonad is sex-limited

**Fig. 1.**
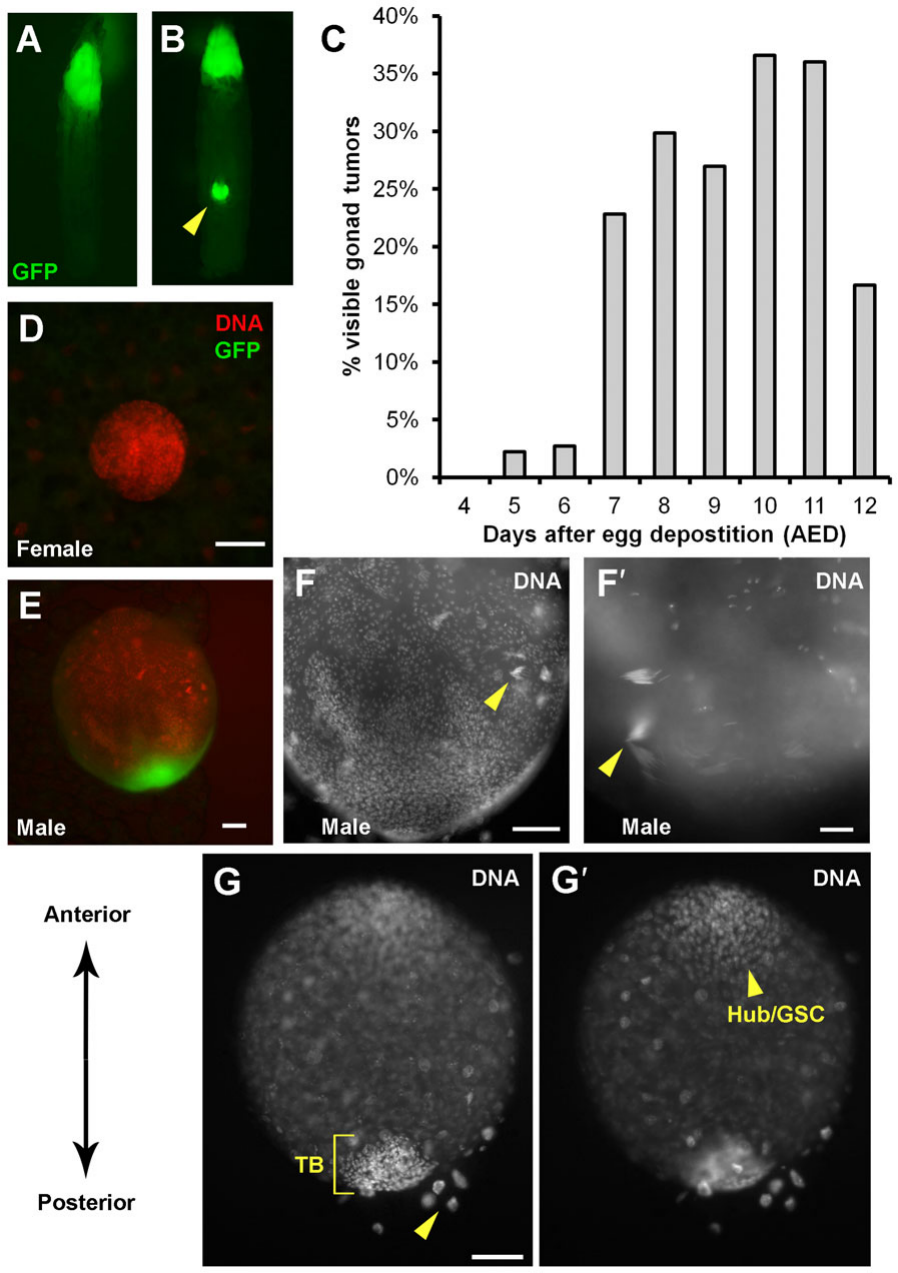
GFP-positive cells grow in gonads of male larvae. Larvae were imaged live for GFP (A,B) or dissected, fixed and stained with a DNA dye before imaging (D–G′). (A,B) GFP-positive growth appears in the cephalic region of all larvae and in the gonads of some larvae (arrowhead in panel B). (C) Quantification of larvae with gonadal GFP. 25 to 149 larvae were removed and counted per day. The average of two independent experiments is shown. (D–G′) GFP is limited to male gonads. Male gonads are larger (D,E), and show signs of spermatogenesis (arrowheads in F,F′) and male-specific cell types: hub cells/GSC at the anterior pole and terminal body (TB) at the posterior pole. (G,G′) Two focal planes of the same gonad. TB cells are more closely packed than hub cells/GSC and can be distinguished by DNA stain. Polyploid fat cells that surround the gonads are also visible (arrowhead in panel G). Scale bar: 50 µm (D,E,F,G,G′), 20 µm (F′).

We generated gonadal tumors using the *eyFLP1* line (Fig. 1A,B). The localization of GFP in gonads was confirmed by examination of dissected tissues as described below. Quantification of tumor incidence over time showed that gonadal GFP was apparent by d7 AED and plateaued at ∼40% of larvae (Fig. 1C). Larvae with uni- or bi-lateral GFP were observed.

Because gonadal GFP occurred in approximately half of the larvae, we investigated whether it was a sex-specific. *Drosophila* gonad development begins as germ cells and somatic gonadal precursors (SGPs) coalesce in the embryo (Casper and Van Doren, 2006). By late 3rd larval instar, sexual dimorphism in gonads is clear (Brown and King, 1961); male gonads were larger and have a more advanced program of gametogenesis (Fig. 1D–F′). In fact, at d7 and later AED, we observed sperm heads with elongated nuclei (Fig. 1F,F′). 3rd instar larvae normally do not display sperm heads; however, prolonged larval life in the presence of neoplastic tumors allowed the gonads to mature and helps us differentiate between male and female gonads unequivocally. DNA stain alone revealed some cell types present in male gonads: hub cells/germline stem cells (GSCs) at the anterior pole, SGPs interspersed with germ cells in the middle, and closely-packed cells of the ‘terminal body’ (TB) at the posterior pole (Fig. 1G,G′) (Casper and Van Doren, 2006; Renault, 2012). The identity of the hub/GSCs, SGPs and TB were confirmed by staining for Eya and Fas3 proteins (supplementary material Fig. S1). Finally, female gonads are smaller and appear homogenous by DNA stain. Using these criteria, we found that all GFP-positive gonads examined were male (*n*>200) while all female gonads examined were GFP-negative (*n*>80). We conclude that gonadal overgrowth due to *Ras^V12^/scrib^1^* mutations is sex-limited, occurring only in males.

### Gonadal overgrowth begins in the terminal body and spreads to the anterior

**Fig. 2.**
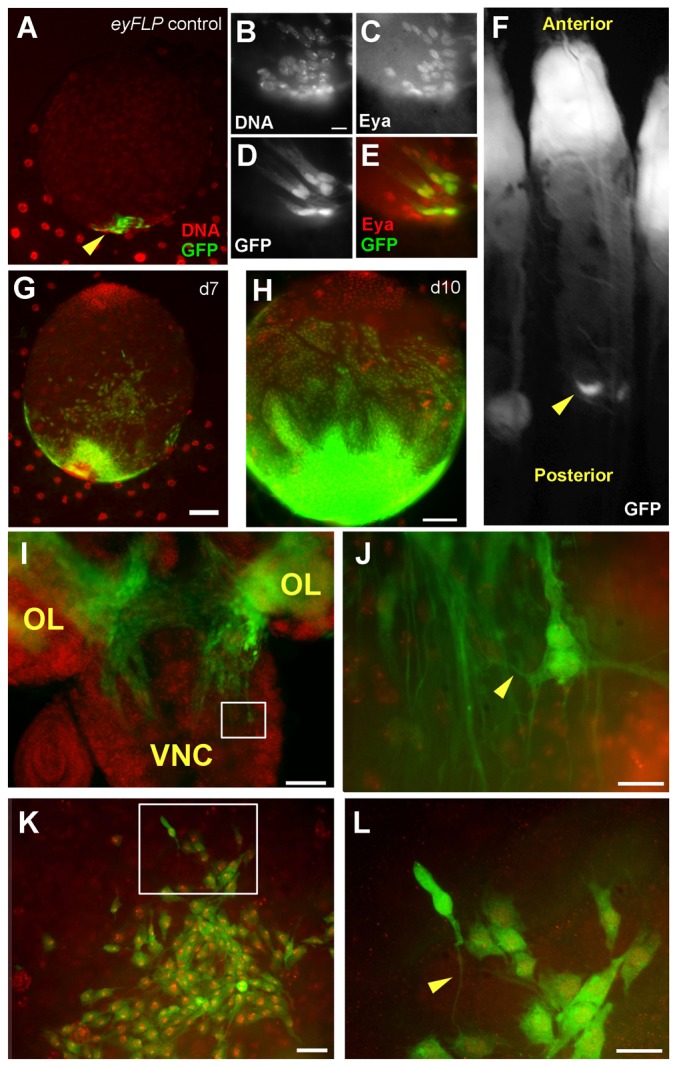
Gonadal neoplasm is likely due to *eyFLP* activity in the terminal body. Larvae were imaged live for GFP (F) or dissected, fixed and stained with a DNA dye and/or antibody to Eya before imaging (the remaining panels and as indicated). (A–E) GFP-positive cells in the gonad originate in the posterior end and are found among cells of the TB. (A) A male gonad from a control (non-tumor-bearing) larva expressing *eyFLP* without UAS-Ras or *scrib^1^* mutation; arrowhead points to densely-packed cells of the TB, some of which show GFP. (B–E) show magnified posterior region of a similar gonad. TB cells are stained with an antibody to Eya in panels C and E. (F) A GFP crescent in a live larva. (G,H) Gonads from tumor-bearing larvae at d7 and d10 AED. The growth of GFP-positive cells is concentrated at the posterior end in both gonads but anterior advancement is greater in the latter. (I–L) Cytoplasmic extensions in cephalic and gonad neoplasms. (I) GFP-positive cells originating in the optic lobe (OL) invade the ventral nerve cord (VNC). The boxed area is magnified in panel J. Arrowheads indicate cytoplasmic extensions. (K,L) GFP-positive cells in the gonad display cytoplasmic extensions. We counted over 20 cells in greater than 5 cephalic and 5 gonad neoplasms. Virtually all GFP positive neoplastic cells neighboring wild-type tissue displayed this phenotype. The area boxed in panel K is magnified in panel L. Scale bar: 50 µm (G,H,I, also applies to A), 10 µm (B, also applies to C–E), 10 µm (J,L), 20 µm (K).

The simplest explanation for male-specificity of gonadal overgrowth is that gonadal *eyFLP1* expression occurs in a cell type(s) present only in male gonads. To test this hypothesis, we examined which gonadal cell type(s) *eyFLP1* is active in. In gonads from *eyFLP1* only controls (without *Ras^V12^/scrib^1^*), GFP-positive cells appeared at the posterior pole where the TB is located (Fig. 2A, arrowhead). Co-staining for Eya confirmed that GFP-positive cells were indeed a subset of TB cells (Fig. 2B–E; supplementary material Fig. S1). The TB is derived from posterior-most SGPs, which are of mesoderm origin; equivalent cells in females are eliminated by apoptosis during embryogenesis (Casper and Van Doren, 2006; Renault, 2012). We conclude that *eyFLP1* is active in a cell type present in male gonads but not female gonads, explaining the observed sex-limited overgrowth.

UAS-GFP appeared in only a subset of TB cells in *eyFLP1* only controls. This was expected because only cells that lost GAL80 through mitotic recombination would be capable of doing so (see Materials and Methods). Likewise, GFP was expressed only in a subset of cells in the eye-antennae disc and the optic lobes in the same larvae (supplementary material Fig. S2). At the points examined, we observed GFP positive cells only in the neuroectoderm and male gonads using this driver.

If *eyFLP1* was active among cells at the posterior pole, we would expect overgrowth to initiate at the same site. Indeed, even in whole live larvae, we saw signs for initiation at the posterior pole as a crescent of GFP facing the anterior (Fig. 2F). Initiation of overgrowth at the posterior and spread to the anterior was also evident in gonads dissected from larvae at d7 and d10 AED (Fig. 2G,H). GFP-positive cells in these gonads expanded beyond the normal location of TB (Fig. 2G versus Fig. 1G), and engulfed the gonad in posterior-to-anterior fashion (Fig. 2H).

*Ras^V12^/scrib^1^* cells in the optic lobe show actin-based cytoplasmic extensions with a proposed role in invasion (Pagliarini and Xu, 2003). We could detect these extensions in GFP images (Fig. 2I,J, arrowhead). Cytoplasmic extensions were also observed in human cells expressing Ras^V12^ and dominant negative scrib^KD^ grown in Matrigel (Dow et al., 2008). We found similar extensions in GFP-positive cells of the gonad (Fig. 2K,L, arrowhead).

Previous authors noted gonadal GFP in some but not all eyFLP lines (Pagliarini and Xu, 2003). This makes it unlikely that gonadal GFP were metastases from cephalic tumors, which would be expected in all eyFLP lines that produced cephalic tumors. In wild-type gonads, the TB remains as a dense packet of cells at the posterior pole during larval stages (e.g. Fig. 1G). In contrast, GFP-positive over-growth exceeded what was seen for the TB in wild-type gonads and appeared to be unrelated to the normal developmental program. Therefore, we refer to gonadal overgrowth also as ‘neoplasia’. Because TB cells are of mesodermal origin and eye discs/optic lobes are of neuro-ectodermal origin, neoplasia in these larvae occurred in cells that originated from different germ layers and were differentiating into different fates.

### Comparative analysis of cephalic and gonad neoplasm

The incidence of neoplastic tumors in different cell types in different organs in the same animal offers a unique opportunity to compare their cell biological characteristics. We examined characteristics that are relevant to oncology: cell cycle checkpoints, apoptosis, aneuploidy and response to a chemotherapeutic drug Taxol.

#### Checkpoints

**Fig. 3.**
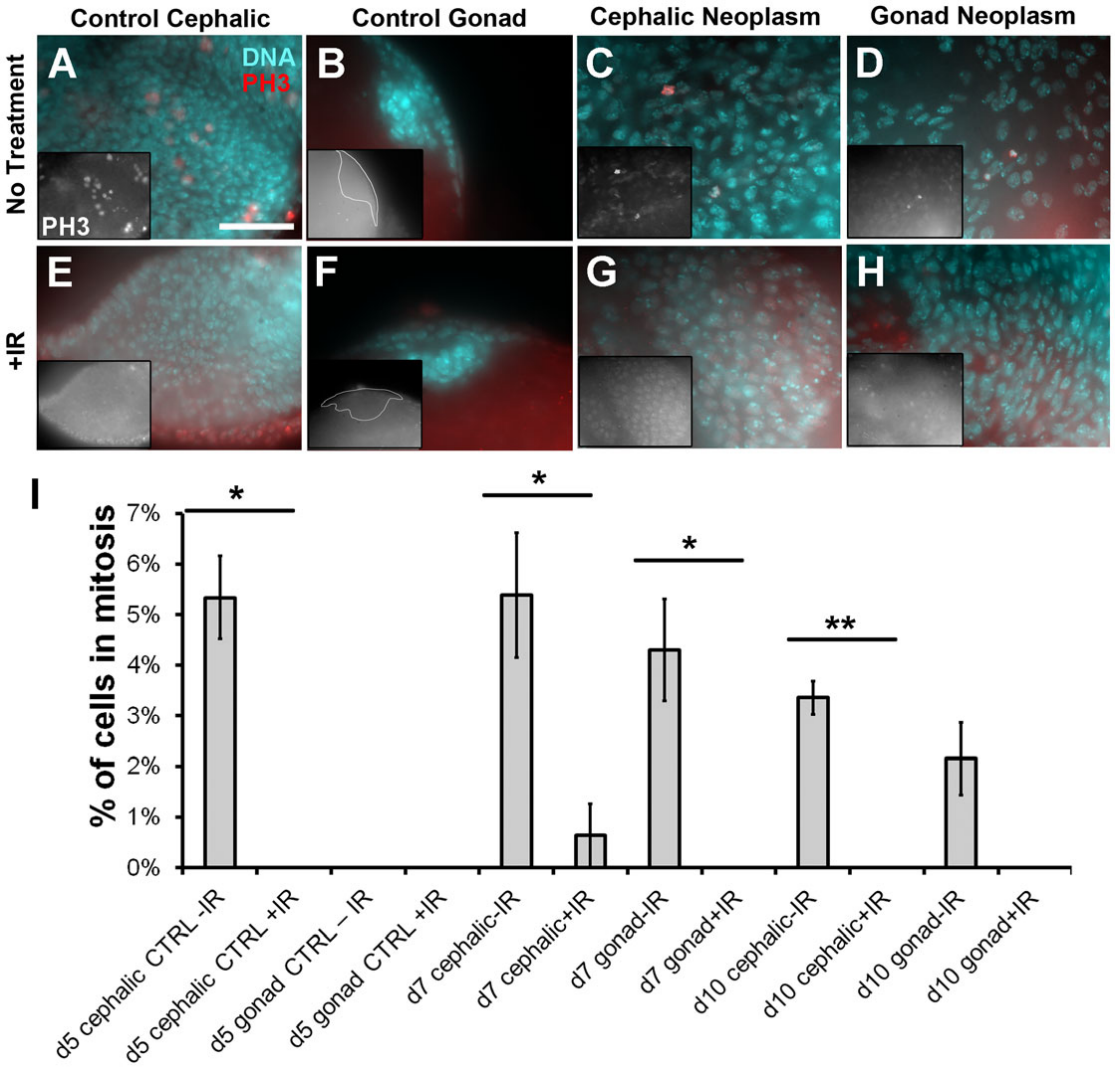
Cephalic and gonad neoplasms maintain an intact DNA damage checkpoint. (A–H) Larvae were irradiated with 4000 R of X-rays 1 hour before dissection, fixed, and stained for DNA (blue) and with an antibody to phospho-histone H3 (pH 3, red). (A,B,E,F) From control cephalic and gonad tissues. (C,D,G,H) From cephalic and gonad neoplasms. Insets in panels A–H show pH 3 only images. (I) Mitotic indices were computed as the number of pH 3-positive cells over the total in panels such as those in A–H. d7 and d10 indicate days AED. No treatment  =  0R; +IR = 4000 R of X-rays. Error bar  =  1 STD. Statistical significance between ±IR pairs was computed using unpaired 2-tailed *t*-test. *P<0.05; **P<0.001. Scale bar: 10 μm (A, also applies to B–H).

In order to examine the DNA damage checkpoint, we exposed larvae to 4000R of X-rays and assayed for mitotic activity one hour later. Irradiated cells with an intact DNA damage checkpoint will halt at G2/M, resulting in a decreased mitotic index compared to un-irradiated controls. 4000R is typically used in *Drosophila* larvae because it is the LD50 for wild-type strains. We used the same dose to facilitate comparison to other studies. Without irradiation, we measured similar mitotic indices in control (non-tumor) eye-antennae imaginal discs and cephalic neoplasms as identified by the expression of the GFP marker (Fig. 3A,C,I). This suggests that neoplastic transformation of cephalic tissues did not speed up the cell cycle. Rather, it may be the prolonged period of proliferation, i.e. the failure to exit the cell cycle and differentiate, that resulted in the overgrowth of cephalic tumors. In contrast, cells of the terminal body of the gonad in control larvae show little or no mitotic activity (Fig. 3B). Upon neoplastic transformation, however, mitotic activity in these cells increased to reach the level found in cephalic tumors (Fig. 3D,I). Thus, in the case of TB cells, neoplastic transformation did increase the rate of proliferation. At one hour after irradiation, we saw significant reduction in the mitotic indices of control cephalic tissues (eye-antennae discs), cephalic tumors and gonadal tumors (Fig. 3E,G–I). We conclude that the DNA damage checkpoint that arrest cells in G2/M is active in cephalic and gonadal tumors alike.

**Fig. 4.**
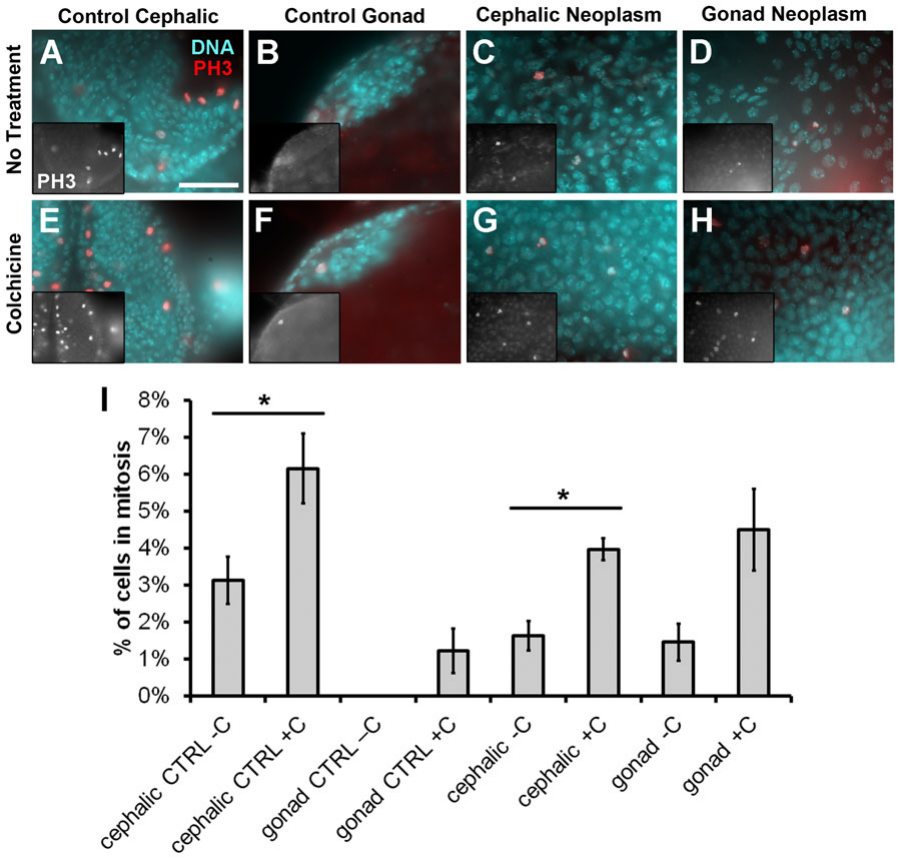
Cephalic and gonad neoplasms maintain an intact mitotic spindle checkpoint. (A–H) Larvae were dissected, incubated in colchicine for 2 hour, fixed, and stained for DNA (blue) and with an antibody to phospho-histone H3 (pH 3, red). (A,B,E,F) From cephalic and gonad control tissues. (C,D,G,H) From cephalic and gonad neoplasms. Insets in panels A–H show pH 3 only images. (I) Mitotic indices were computed as the number of pH 3-positive cells over the total in panels such as those in A–H. No treatment  =  PBS only; colchicine  =  125 µM. Error bar  =  1 STD. Statistical significance between ±C pairs was computed using unpaired 2-tailed *t*-test. *P<0.05. Scale bar: 10 μm (A, also applies to B–H).

In order to examine the spindle assembly checkpoint, tissues were incubated with the microtubule depolymerizer colchicine for 2 hours. Without microtubules, cells with an intact spindle checkpoint will accumulate in mitosis, increasing the mitotic index. Mitotic indices in GFP-positive cephalic and gonad neoplasms showed a similar increase of about 3-fold compared to controls without colchicine (Fig. 4C,D,G–I). This is in agreement with the increase in mitotic activity of about 2-fold we saw in control brains and gonads (Fig. 4A,B,E,F,I). We note that basal mitotic indices here (without colchicine) differed from those in radiation experiments (without IR), possibly because the samples had been incubating in medium for 2 hour before fixing, whereas they were fixed immediately after dissection in radiation experiments. We conclude that the spindle assembly checkpoint that arrests cells in mitosis is intact in cephalic and gonad neoplasms.

#### IR-induced apoptosis

**Fig. 5.**
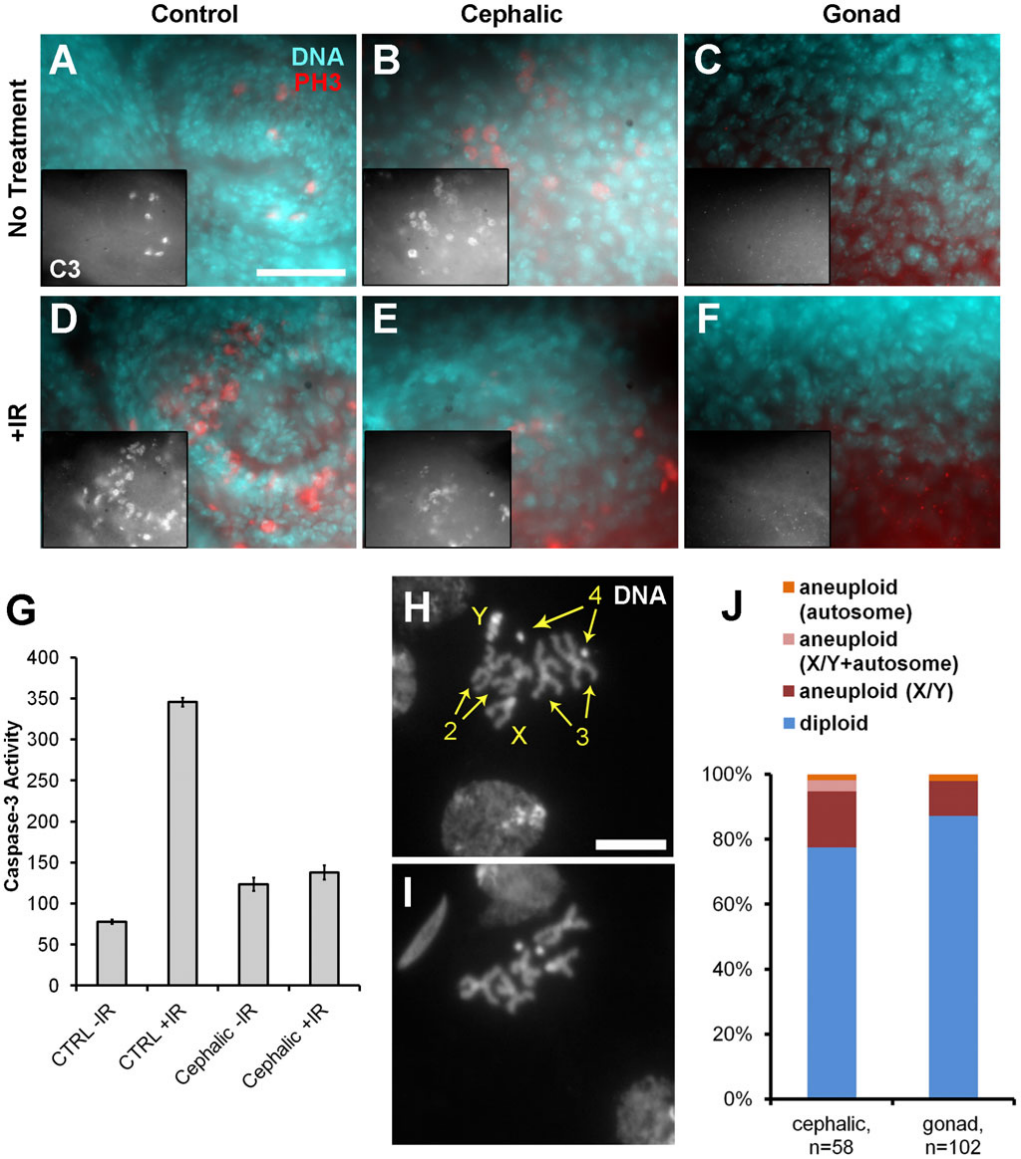
Cephalic and gonad neoplasms show resistance to IR-induced apoptosis and increased aneuploidy. (A–F) Larvae were dissected 4 hour after irradiation with 4000 R of X-rays 4 hour, fixed, and stained DNA (blue) and with an antibody to activated caspase-3 (C3, red). (A,D) From wild-type imaginal wing discs in larvae with cephalic neoplasms. (B,C,E,F) From cephalic and gonad neoplasms. We examined between 5 and 10 cephalic and gonad neoplasms with the same trend of high and low basal apoptosis, respectively. Insets in panels A–F show caspase-3 only images. (G) Activated caspase-3 levels were measured in control tissues and cephalic neoplasms using an *in vitro* caspase-3 activity assay. Units are in fluorescence and normalized to the protein levels in each lysate. (H,I) Examples of chromosomes from gonads stained for DNA. Four pairs of *Drosophila* chromosomes are indicated in panel H. Panel I is missing a sex chromosome. (J) Aneuploid mitoses are expressed as % of total. The difference between cephalic and gonad neoplasm is not significant (*P* = 0.12, 2-tailed Fisher's exact test). Scale bar: 10 μm (A,H; also applies to B–F,I).

Apoptosis is a major response to ionizing radiation (IR) and accounts in part for the therapeutic effect of radiation therapy in cancer. To examine whether cephalic and gonad neoplasms can undergo IR-induced apoptosis, we irradiated the larvae with 4000R of X-rays, fixed, and stained the tissues for cleaved active caspase-3. Wing imaginal discs from tumor-bearing larvae served as a control in these experiments and showed very little caspase-3 staining without irradiation (Fig. 5A). At 4 hr after irradiation, wing imaginal discs showed increased caspase-3 staining as expected (Fig. 5D). This increase was confirmed by *in vitro* caspase-3 activity assays (Fig. 5G). Without irradiation, cephalic neoplasms showed significant caspase-3 staining (Fig. 5B) and caspase-3 activity in *in vitro* activity assays (Fig. 5G) using dissected GFP-marked cephalic neoplastic tissues. Interestingly, there was no significant increase in caspase-3 staining (Fig. 5E) or activity (Fig. 5G) after irradiation in cephalic neoplasms. We conclude that cephalic neoplasms are defective for IR-induced apoptosis. The gonad neoplasms showed no constitutive caspase-3 staining and no increase in this signal after IR (Fig. 5C,F). The neoplastic tissue in the gonads was of lower abundance and harder to isolate cleanly; we were unable to obtain sufficient material for *in vitro* caspase-3 activity assays. We conclude that even though cephalic neoplasms appear to have a high basal rate of apoptosis and gonad neoplasms have virtually none before treatment, neither responded to IR. The proapoptotic protein Hid is a homolog of mammalian SMAC/DIABLO proteins and plays an essential role in IR-induced apoptosis in *Drosophila*. Reduction of *hid* gene dosage by half is enough to prevent IR-induced apoptosis (Brodsky et al., 2004). Hid is inhibited through phosphorylation by MAPK (Bergmann et al., 1998). We speculate that in RAS-driven tumors such as those under study here, MAPK activity inhibits Hid and prevents IR-induced apoptosis.

#### Aneuploidy

Using mitotic chromosome spreads (Fig. 5H,I), we detected a low but significant level of aneuploidy, a hallmark of human cancers, in both cephalic and gonad neoplasms (Fig. 5J). All instances of aneuploidy involved chromosome loss, not gain. Sex chromosomes were most frequently affected; 92% of aneuploid cells in cephalic tumors and 85% of aneuploid cells in gonad tumors had a single X without accompanying X or Y. Wild-type brains, in contrast, have an undetectable level of aneuploidy in these assays (Stumpff et al., 2004; data not shown). Constitutively active Ras can induce aneuploidy and chromosomal instability in cultured cells within as few as one cell cycle (Denko et al., 1994; Guerra et al., 2003; Woo and Poon, 2004). The mechanism for Ras-induced aneuploidy remains elusive, but our results indicate that this activity of oncogenic Ras is conserved in *Drosophila*.

#### Sensitivity to Taxol

**Fig. 6.**
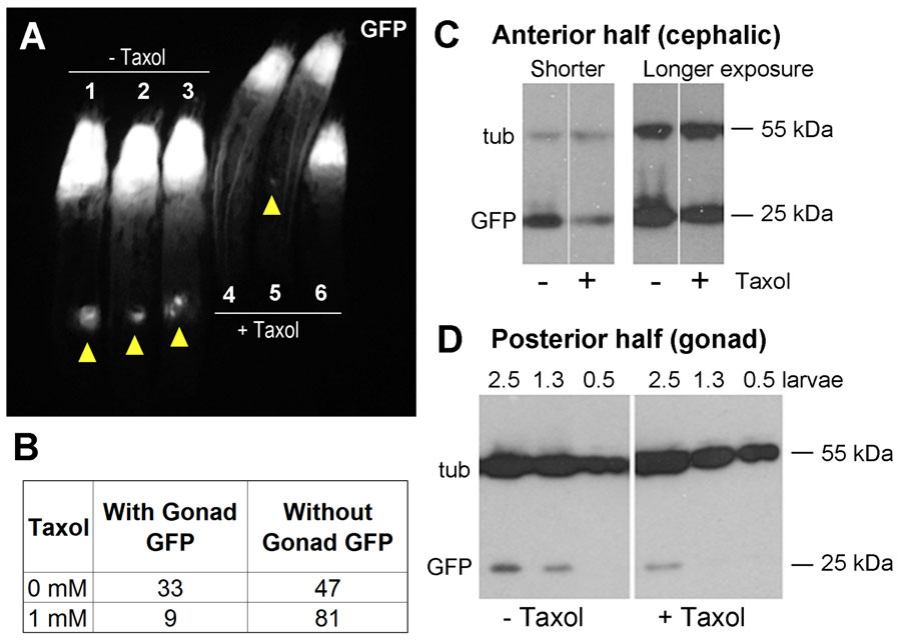
Cephalic and gonad neoplasms are reduced after treatment to Taxol. (A) Larvae were imaged live for GFP on d9 AED. Arrowheads indicate GFP-positive gonads. Quantification of the GFP fluorescence in this image using Image J software showed that Taxol treatment reduced the mean cephalic GFP from 126±12 (mean fluorescence signal arbitrary units, −Taxol) to 81±8 (+Taxol). The difference was significant (*P*<0.01, 2-tailed Student's *t*-test). Similarly, Taxol treatment reduced the mean gonadal GFP from 24±7 (−Taxol) to 8±3 (+Taxol). The difference was also significant (*P*<0.05, 2-tailed Student's *t*-test). (B) The reduction of gonadal GFP by Taxol was significant (*P*<10^−5^, Fisher's exact test, 2-tail). (C,D) Western blots of extracts from anterior (C) or posterior halves (D) of Taxol-treated larvae compared to control vehicle-treated larvae as in panel A. (C) Tubulin signal is the same in −/+ Taxol lane but GFP is reduced in the +Taxol lane. Two different exposures are shown. In panel D, GFP signal is much weaker than the tubulin signal, therefore we loaded ‘larval equivalents’ (see Materials and Methods) and used the tubulin signal to confirm equal loading. For example, 2.5  =  extracts from 2.5 larvae; 1.3  =  extracts from 1.3 larvae. Molecular weight markers are shown along the side. Similar data were obtained in two independent experiments.

We used a protocol that was used successfully in this tumor model to identify small molecules with therapeutic potential (Willoughby et al., 2013). We administered Taxol at d4 AED and examined the larvae daily up to d10 AED. Taxol-treated larvae were similar in size to untreated controls, suggesting that Taxol concentrations used here did not prevent growth generally. Taxol, however, reduced GFP in the cephalic region (Fig. 6A; see figure legend for quantification). Treated animals also appeared healthier and more active; of all larvae that were briefly chilled to immobilize and lined up for imaging in Fig. 6A, untreated larvae remained flaccid whereas treated larvae recovered mobility and were crawling away by the time the image was acquired. None were rescued to pupa or adult stages, however. Taxol also reduced or eliminated gonadal GFP (Fig. 6A, arrowheads, Fig. 6B). In this protocol, Taxol was added before gonadal GFP appeared (Fig. 1C). Therefore, Taxol may have been able to prevent new growth or growth at early stages in the gonad. Cephalic GFP was already visible at d4 AED, which may have allowed Taxol to reduce but not completely eliminate the tumors.

Regardless, both cephalic and gonad neoplasms, we conclude, were sensitive to Taxol. These results were confirmed by Western blotting for GFP in control and Taxol-treated larvae (Fig. 6C,D). Colon cancer cells expressing oncogenic KRas are hypersensitive to Taxol compared to isogenic wild-type cells (Luo et al., 2009). Our results indicate that this characteristic of Ras-driven cancer is also conserved in *Drosophila*.

In conclusion, we found that neoplasms induced by *Ras^V12^/scrib^1^* mutations in tissues of neural ectodermal and mesodermal origins behaved similarly in every manner examined: cell cycle checkpoints, cellular morphology, apoptosis, aneuploidy and response to Taxol. As stated above, oncogenic mutations in different tissues could produce one of two outcomes. Oncogenic mutations may exert their effect regardless of cell type differences. Alternatively, underlying differences among tissues could interact with oncogenic mutations to produce different outcomes. We conclude that, at least for *Ras^V12^/scrib^1^* and the tissues examined here, oncogenic effects appear to override tissue-specific differences to produce neoplasms with similar mitotic indices, resistance to IR-induced apoptosis, increased aneuploidy, and sensitivity to a chemotherapeutic agent. The system described here has been used to generate tumors by combining *scrib* mutations also with oncogenic Notch or Abrupt transcription factor, instead of Ras^V12^ (Brumby et al., 2011; Brumby and Richardson, 2003; Turkel et al., 2013). It would be interesting to examine other tissues and oncogenic mutant combinations using various drivers, to see if the override of tissue-specific differences by oncogenic transformation is universal.

## MATERIALS AND METHODS

### Drosophila

All stocks used here have been described before (Pagliarini and Xu, 2003): *eyFLP1*; Act>y^+^>Gal4, UAS-GFP; P[FRT82B], *Tub-GAL80* virgin females were crossed to w; *UAS-Ras^V12^*; P[FRT82B], *scrib^1^*/TM6B males to generate tumors and to P{ry[+t7.2] = neoFRT}82B ry[506] males to generate controls that express GFP in the gonad (Fig. 2A). *y^1^w^1118^* served as wild type. Embryos were collected on Nutri-fly (Bloomington Formula), and cultured at 25°C.

### Staining

Larvae were dissected in PBS and fixed in 10% formaldehyde in PBT (PBS + 0.2% Tween-20) for 10 minutes at room temperature (RT; for pH 3) or in 4% paraformaldehyde in PBTx (PBS + 0.1% Triton X-100) for 30 minutes at RT (for Caspase-3, Eya, and Fas3). For antibody staining, samples were blocked in 3% normal goat serum (NGS) in PBT (for pH 3) or 5% NGS in PBTx (for Caspase-3, Eya, and Fas3) for at least 1 hour before incubation with primary antibodies: rabbit anti-pH 3 Ser10 (1:1000, Upstate Biotech), rabbit anti-Caspase-3 (1:100, Cell Signaling cat. no. 9661 lot 32), mouse monoclonal anti-Eya and anti-Fas3 (1:25, Developmental Hybridoma Bank), in block for at least 1 hour at RT. Secondary antibodies anti-rabbit rhodamine, anti-mouse FITC or anti-mouse Rhodamine Red-X (Jackson) diluted 1:500 in block. Samples were stained with 10 µg/mL Hoechst33258 (Sigma) in PBT or PBTx and mounted in Fluoromount G (Southern Biotech).

### *In vitro* caspase-3 assay

Caspase-3 activity was measured using the Caspase-3/CPP32 Fluorometric Assay Kit (BioVision) according to the manufacturer's protocol. Tissues were dissected in chilled PBS and stored at −80°C. Fluorescent readings were adjusted based on the lysate protein concentration, which was measured using a DC Protein Assay (Bio-Rad).

### Chromosome squashes

Chromosome squashes were performed as described before (Pimpinelli et al., 2010), with a 1.5 hour incubation in saline (0.7% w/v NaCl in water) containing 1.5 mM colchicine (Sigma) to trap mitotic cells. The samples were stained with Hoechst33258 as described.

### Imaging

Whole larvae were imaged for GFP using a Nikon SMZ 1500 stereomicroscope. Fixed tissues were imaged using a Leica DMR compound fluorescence microscope. All images were acquired at room temperature. Objective lenses used were 5×air/NA 0.15, 10×air/NA0.30, 20×air/NA0.50, 40×air/NA0.74 and 100×oil/NA1.30. Images were collected using a SensiCam CCD camera and Slidebook software (Intelligent Imaging). Slidebook images were exported as TIFF documents, processed in Photoshop (Adobe) and assembled in Illustrator.

### Taxol treatment

200 µl of water containing 2 µl of DMSO (control) or 2 µl of 100 mM Taxol (Paclitaxel, Sigma) in DMSO was added per culture vial, and gently mixed into the top layer of food containing larvae using a pipet tip. Each vial contained approximately 5 ml of food. Lower Taxol concentrations administered in this manner did not change tumor size.

### Western blotting

Larvae were bisected to separate the head and gonad tumors and flash frozen in liquid nitrogen. Larvae were homogenized in PBS, an equal volume of 2× SDS loading buffer was added to the extract, and boiled to denature proteins. The extracts were separated on 10% polyacrylamide gels and Western blotted using standard protocols. Primary antibodies were 1:500 rabbit anti-GFP (Life Technologies cat. no. A11122) and 1:100 mouse anti-tubulin (Developmental Hybridoma Bank) in block (PBT with 0.2% Tween-20, 5% milk). HRP-conjugated secondary antibodies (Amersham) were used at 1:2500 in block. Western blots were developed using ECL (Thermo Scientific). From the known larval equivalent in the final sample (e.g. 1 larva/4 µl), various amounts of the sample were loaded to give the larval equivalents shown on the figures (e.g. loading 2 µl would give 0.5 larva equivalent).

## Supplementary Material

Supplementary Material
