# Supplementary material for: Oncogenic mutations produce similar phenotypes in Drosophila tissues of diverse origins
Source: Biol Open. 2014 Feb 25;3(3):201–9. doi: 10.1242/bio.20147161 (PMC4001236; doi:10.1242/bio.20147161)
Supplement: Supplementary Material [file supp_3_3_201__index.html]

Oncogenic mutations produce similar phenotypes in Drosophila tissues of diverse origins — Supplementary Material 

# Oncogenic mutations produce similar phenotypes in *Drosophila* tissues of diverse origins

## bio.20147161 Supplementary Material

**Files in this Data Supplement:**

- Supplementary Material - Stefanie Stickel and Tin Tin Su doi: 10.1242/bio.20147161
