# Supplementary material for: Oncogenic mutations produce similar phenotypes in Drosophila tissues of diverse origins
Source: Biol Open. 2014 Feb 25;3(3):201–9. doi: 10.1242/bio.20147161 (PMC4001236; doi:10.1242/bio.20147161)
Supplement: Supplementary Material [file supp_bio.20147161_bio.20147161-s1.pdf]

Biology Open (2014) 000, 1–8 doi:10.1242/bio.20147161

Supplementary Material
Stefanie Stickel and Tin Tin Su **doi: 10.1242/bio.20147161**

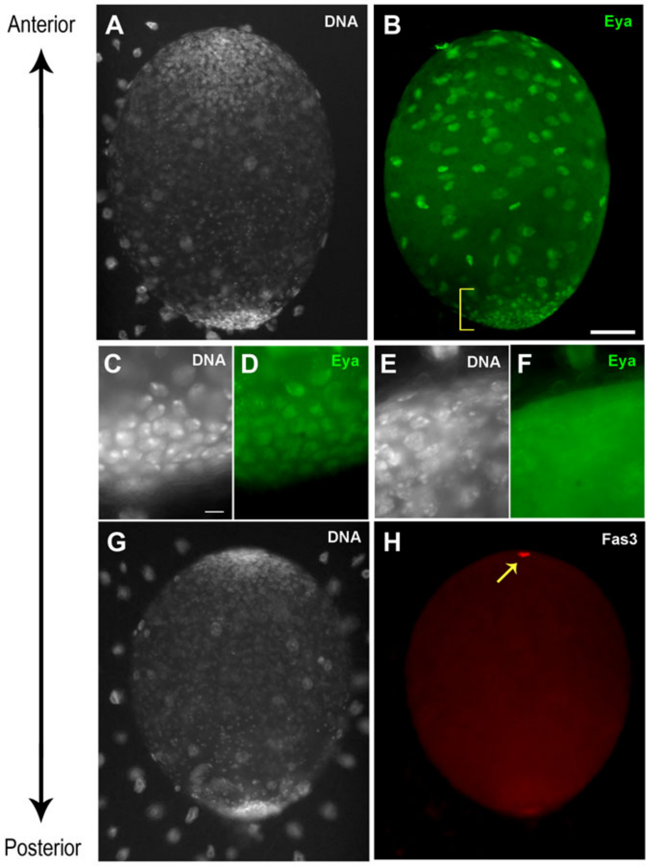

Fig. S1. Eya and Fas2 expression in cells of the male gonad. Gonads were dissected from male wandering 3rd instar y^1w^{1118} larvae, fixed and stained with antibodies against Eya (B,D,F) or Fas3 (H) and for DNA (A,C,E,G). Somatic Gonadal Precursors (SGPs) including a subset that forms the terminal body (yellow bracket) stain for Eya in (B, magnified in D). Hub cells and germline stem cells (GSCs) at the anterior pole do not show Eya stain (E,F). Instead, hub cells stain strongly for Fas3 (H, arrow). Scale bar: 50 µm (A,B,G,H), 5.5 µm in C–F.

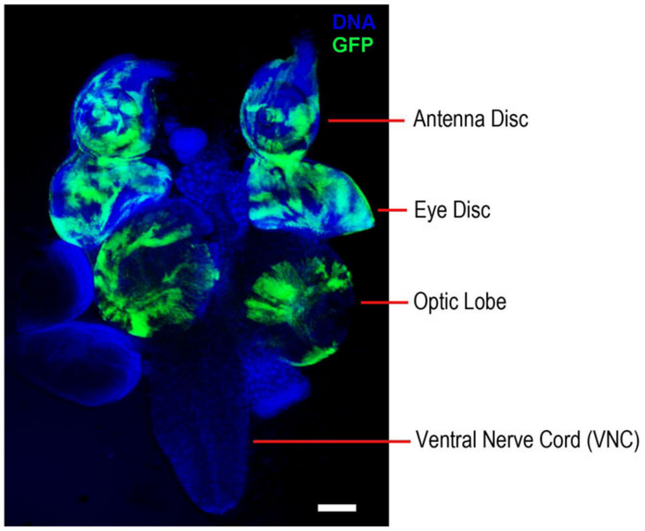

Fig. S2. Cephalic tissues that express GFP from *eyFLP1*. Tissues shown were dissected from 3rd instar larvae from a control *eyFLP1* only cross (see Materials and Methods), fixed and stained for DNA. Bilateral expression of GFP is seen in eye antennae discs and the optic lobes but not the ventral nerve cord. Note the patchy expression of GFP as expected from clonal induction through mitotic recombination. Scale bar: 100 µm.
